# Supplementary material for: Optimization of COVID-19 vaccination and the role of individuals with a high number of contacts: A model based approach
Source: PLoS One. 2022 Mar 8;17(3):e0262433. doi: 10.1371/journal.pone.0262433 (PMC8903293; doi:10.1371/journal.pone.0262433)
Supplement: S1 File — (ZIP) [file pone.0262433.s001.zip › suporting.pdf]

Optimization of COVID-19 vaccination and the role of individuals with a high number of contacts: a model based approach – Supporting information

Epidemiological model

The model delayed system of ordinary differential equations corresponding to the flow chart in Figure 4 of the main text are given by:

$$
\begin{aligned}
&\frac{dS_i}{dt} = -\lambda_i S_i - \mu' S_i + \kappa' \delta_{i,1} - \rho_i^{(S)}(t), \qquad \frac{dE_i}{dt} = \lambda_i S_i - \sigma E_i - \mu' E_i,\\
&\frac{dI_i}{dt} = (1-\chi)\sigma E_i - \gamma I_i - (1-\chi)\zeta_i \sigma E_i(t-\tau_1) - \mu I_i',\\
&\frac{dA_i}{dt} = \chi \sigma E_i - \gamma A_i - \mu A_i',\\
&\frac{dH_i}{dt} = -\psi H_i + (1-\chi)\zeta_i \sigma E_i(t-\tau_1) - (1-\chi)\theta_i \zeta_i \sigma E_i(t-\tau_2) - \mu H_i',\\
&\frac{dR_i}{dt} = \gamma I_i + \gamma A_i + \psi H_i - \mu R_i' - \rho_i^{(R)}(t),\\
&\frac{dV_i}{dt} = e_v \rho_i(t) - \mu V_i', \qquad \frac{dU_i}{dt} = (1-e_v)\rho_i(t) - \mu U_i',\\
&\rho_i(t) = \rho_i^{(S)}(t) + \rho_i^{(R)}(t),
\end{aligned} \tag{S1}
$$

where all variables are taken at time t except when otherwise shown, $\rho_i^{(S)}(t)$ and $\rho_i^{(R)}(t)$ are the number of vaccines (comprising the required number of doses for immunization) divided by N_0, at time t, given to the susceptible and recovered individuals, respectively, and e_v the efficacy of the vaccine. For economy of space and notation we kept the aging rates between age groups implicit. The different variables are proportions with respect to the initial population and given in table 2 of the main text. The force of infection in Eq. (S1) is given by

$$\lambda_i = \sum_{j=1}^{M} \beta_{i,j}(I_j + \xi A_j)/n_i, \tag{S2}$$

with $\beta_{i,j}$ the components of the transmission matrix. It is worth showing how this expression is obtained. We start by noting that $\lambda_i S_i N \delta t$ gives the total number of susceptible individuals infected during the (small) time interval δt. The contact matrix with elements $C_{i,j}$ is defined as the average number of contacts per unit of time of an individual of age group j with any individual of age group i. The proportion of such contacts with susceptible individuals is $S_i N_0/N_i$ (N_i being the population of age group i). The total number of contacts between all infected (I_j) individuals of any age group with susceptible individuals of age group i during the time interval δt is given by $\sum_j (S_i N_0/N_i) C_{i,j} I_j \delta t$. Now denoting the probability of transmission per contact by p_c and supposing it is independent of which age groups are interacting, we obtain that

$$\lambda_i S_i \, N_0 \delta t = \sum_{j=1}^{M} p_c S_i \frac{N_0}{N_i} C_{i,j} I_j N_0 \delta t. \tag{S3}$$

To add the contribution of asymptomatic individuals we replace I_j by $I_j + \xi A_j$ in this last equation, and then obtain the expression in Eq. (S2) by identifying $\beta_{i,j} = p_c C_{i,j}$.

The numeric solution for the model equation in Eq. (S1) is implemented in the low level C language using a Runge-Kutta fourth order integrator. All scripts and programs are written in the Maple computer algebra package. Since the present model is essentially a mean-field simplification of the more complex phenomenology of an epidemic spread, and due to the time-delay in the equations, we avoid negative variable values by properly testing for positivity at each time step. Otherwise, the system equations have all the required consistency properties.

We write that $C_{i,j}(t) = \omega(t) C_{i,j}(0)$, with $C_{i,j}(0)$ the components of the contact matrix prior to the pandemic, and $P(t) \equiv \omega(t) p_c$ and thus $\beta_{i,j} = P(t) C_{i,j}(0)$. The determination of $P(t)$ is discussed below

Contact matrix

Up to the authors knowledge no such study was performed for either Portugal or Brazil. This difficulty can be overcome by considering that the social contacts structure of the European countries in Ref. [39] is similar to that in Portugal as the dispersion for the contact matrices for the countries considered in this study is small, and it is quite reasonable to use then as an estimate for other European countries. For Brazil, where the great majority of the population also live in urban centers, the same supposition stands. This can me better justified by noting that for countries with similar economic structures and cultural setups the number of contacts $C_{i,j}$ does not depend significantly on the total population of the country, and is mainly determined by the average over different types of activities of each person (school, work, transportation, etc. . .). We then compute the average contact matrix for the eight countries in the study of Ref. [39] and properly joining the different age-groups. This results in an estimate of the contact matrix that is a proper approximation for the purposes and scope of the present study, and is shown as a heat map in Fig. S1.

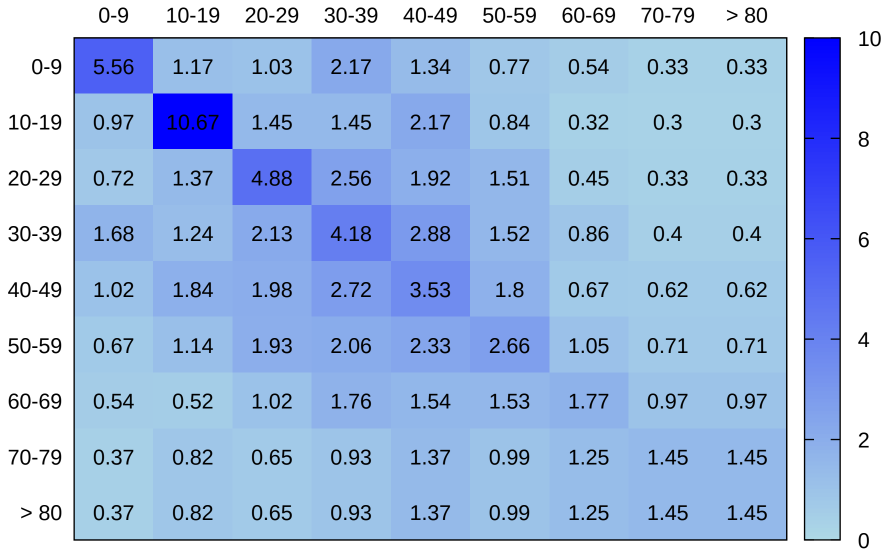

Fig S1. Heat map for the estimate of the contact matrix. The entries give the number of contacts per day.

Fitting $P(t)$ from data

The model is calibrated by fitting its output to the actual number of total deaths. The transmission matrix is obtained as the product of the multiplied contact matrix and a time-dependent transmission probability P(t). By using a step function for this probability, with 21-day intervals, we minimize the mean square deviation of the time series for the number of deaths over the last seven days and the model output for the same quantity. The time-dependent parameter $P(t)$ is obtained from a numeric fit of the output of the numeric solution of the model equations. We chose as fitting function one with constant values on 21 days intervals, with values that can vary from interval to interval. The length of the interval was chose on a trial and error basis in order to yield a good fit and without a too great numeric effort. The constant values at each interval are obtained by minimizing the quadratic error:

$$\mathcal{E} = \sum_{n=1}^{N_{days}} \left[\Delta_a(n) - \Delta_{mod}(n)\right]^2 , \tag{S4}$$

where $\Delta_a(i)$ and $\Delta_{mod}(i)$ are the actual and fitted number of deaths over the last 7 days at day n, respectively, and N_{days} is the total number of days considered for the fitting. We used a simplex method for the function minimization. Figure S2 shows the real and fitted data for Portugal and Brazil, evidencing the good quality of the fit.

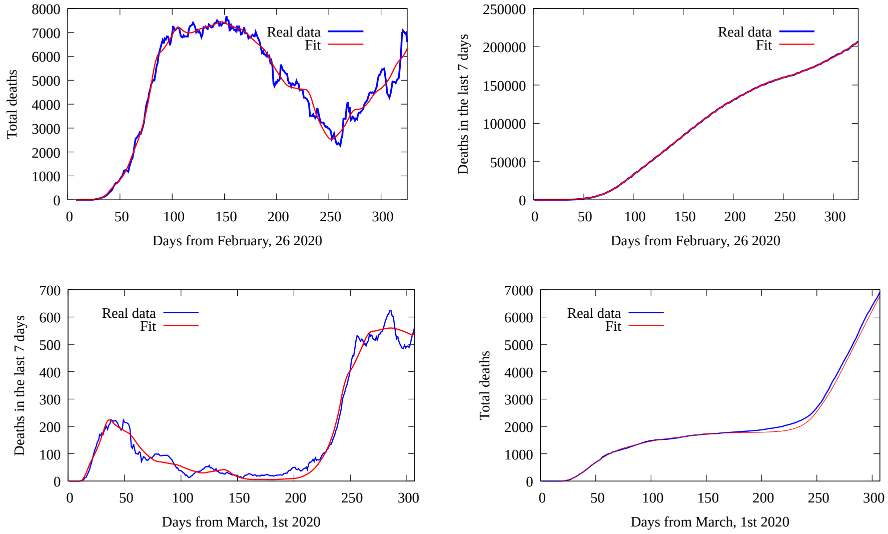

Fig S2. Actual and fitted number of deaths on the last 7 days and total deaths by COVID-19 for Brazil (top) and Portugal (bottom).

Parameter	Definition	Value (IC 95%) [Ref]
ψ	Recovery rate from hospitalization	$1/17.5$ days^{-1} [8,33]
σ	Inverse of incubation time	$1/5.0$ days^{-1} [34]
γ	Recovery rate for non hospitalized individuals	$1/3.69$ days^{-1} [35]
θ_i	Fatality rate among hospitalized individuals	$L_i^{(0)}/\zeta_i$
τ_1	Median time from first symptoms to hospitalization	3.3 [34]
τ_2	Average time from first symptoms to death	16.8 [8]
χ	Proportion of asymptomatic cases	17.9% [36]
ξ	Contagiousness of asymptomatic individuals with respect to symptomatic individuals	55% [35]

Table S1. Parameters in the epidemiological model. The index i refers to the age group. In the absence of an index the parameter has the same value for all age groups. $L_i^{(0)}$ and ζ_i are given in Tables S2 and S3.

0 – 9	10 – 19	20 – 29	30 – 39	40 – 49	50 – 59	60 – 69	70 – 79	≥80
0.0%	0.2%	0.2%	0.2%	0.4%	1.3%	3.6%	8.0%	14.8%

Table S2. Infection fatality ratio $L_i^{(0)}$ according to age group [33].

0 a 9	10 a 19	20 a 29	30 a 39	40 a 49	50 a 59	60 a 69	70 a 79	80 or more
0%	0.408%	1.04%	3.43%	4.35%	8.16%	11.8%	16.6%	18.4%

Table S3. Hospitalization probability ζ_i for each age group [33].
