# Supplementary material for: Optimization of COVID-19 vaccination and the role of individuals with a high number of contacts: A model based approach
Source: PLoS One. 2022 Mar 8;17(3):e0262433. doi: 10.1371/journal.pone.0262433 (PMC8903293; doi:10.1371/journal.pone.0262433)
Supplement: Attachment — Submitted filename: rebuttal.docx [file pone.0262433.s002.docx]

**Rebuttal Letter**

Dear M. Shamim Kaiser, PhD
Academic Editor of PLOS ONE

Please find below the authors answers to the Academic Editor, the Journal, and the Rederees Comments.

Sincerelly Yours,

Prof. Tarcísio M. Rocha Filho

On behalf of all authors.

**ACADEMIC EDITOR:**

The following major issues must be addressed:

COMMENT: "Could you justify why the particular age group was picked for the model development?"

ANSWER: The different age groups were chose based on the available information by age group on hospitalization and death probabilities by infection. The age group for superspreaders correspond to the the "average" age group pf the economically active population, and is a simplification introduced in the model. In this way, we believe it depicts well what occurs with those individuals with a much greater number of contacts in the whole population.

COMMENT: "The authors only chose Brazil and Portugal. They are in different phases of vaccination. In sensing the full data, my first notice is that Brazil fitting into the unpredictable model is just 20.3 percent compared to Portugal. It would be amazing if the authors recognized that the vaccination stage is over 50% or more other than Brazil and Portugal."

ANSWER: We added a comment on this in the last paragraph of the Discussion section.

COMMENT: "I have noticed some typos and grammatical issues, thus read the article carefully."

ANSWER: we did it.

**Journal Requirements:**

We correctec the funding information and added to it the phrase "The funders had no role in study design, data collection and analysis, decision to publish, or preparation of the manuscript." The complete funding statemente is given in the cover letter.

COMMENT: "5. Please upload a copy of Figure S3, to which you refer in your text on pages 5 and 6. If the figure is no longer to be included as part of the submission please remove all reference to it within the text."

ANSWER: There is no citation to Figure S3, but to Table S3, which is included in the supplemental material.

COMMENT: "PLOS authors have the option to publish the peer review history of their article (what does this mean?). If published, this will include your full peer review and any attached files."

ANSWER: Yes

**Reviewers' comments:**

**Reviewer #1**

COMMENT: "This manuscript is well written but needs correction of some important points before publication. Like in introduction need to add information from the below recent papers;

https://doi.org/10.3390/vaccines9080864

https://doi.org/10.3390/vaccines9050416"

ANSWER: We added a comment on the need to consider willingness to vaccinate and the economic cost for individuals, and cited the suggested references.

COMMENT: "There is not enough methodological information in the abstract."

ANSWER: We added some information of the methodology in the abstract

COMMENT: "Present the methods section before the results and discussion."

ANSWER: Done.

COMMENT: "Could you please justify the reasons why the specific age group was selected for the model development and what are the reasons for vaccination failure among that group.

Add more clarity around super spreaders group."

ANSWER: We explained more explicitly in the first paragraph of the subsection "Implementing the superspreaders group".

COMMENT: "The strengths of the research are not clearly established."

ANSWER: We added a paragraph in the end of the Discussions section. We believe that this will put more clearly the strength and goals of

the present work.

**Reviewer #2**

COMMENT: "The authors have chosen only Brazil and Portugal as they are originated from these countries. These they are in different vaccination stages, with 20.3% and 64.75% of the population in Brazil and Portugal fully vaccinated, respectively. By sensing the whole study, my first observation is that the case of Brazil fitting in the model given unseemly results in compare with Portugal as Brazil has only 20.3%. Its troublesome to discuss future optimal vaccination campaigns for any community by model fitting only a small population like Brazil. It would be great if the authors considered another besides Brazil and Portugal where the vaccination stage is above 50% or more."

ANSWER: The present work is a first study in this direction, and is a proof of concept for the need of a more detailed consideration of different possibilities for vaccination strategies. A more detailed study, with a version of the model considering different kinds of vaccine is under way, mainly for countries with a very low proportion of the population fully vaccinated and located mainly in sub-Saharan Africa. We hope that the current approach may be useful in countries where the available number of doses is still very small.

COMMENT: "How does the authors deal with the uncertainties of the model. There are many considerations the authors considered to run the model. For example, reinfection proportion is negligible and that mixing is homogeneous, discarding heterogeneous effects in the population. Why and How?"

ANSWER: As for any modeling approach, some simplifications are considered, while more important aspects are considered with more care. Heterogeneous effects may be considered in more complex models, but are also very hard to model as more information is needed. The use of homogeneous mixing, although being a simplification often used, allows to calibrate the model with available data and yet obtain important insights on the pandemic dynamics. Reinfection is expected to be not significant for the amount of time considered in the present work.

COMMENT: "How does the authors consider that 20% of the age group from 30 to 39 years of age are superspreaders, which amounts to 3.2% of the total population of Brazil and 2.5% of Portugal?"

ANSWER: We introduced some explanation on this on the first paragraph of the subsection on "Implementing the superspreaders group".

COMMENT: "Different age groups (9 groups here) should have different behavioral pattern and it varies with locality and time. Why didn’t the authors use any coefficient or else to remove the uncertainties raised the by the said issues?"

ANSWER: This is indeed true. The dependence on locality is obtained mainly by field studies such as the one in Mossong et al. Unfortunately no such study was performed for Brazil or Portugal, so we have to assume that the average number of contacts between two age-groups is the same as the average for the European countries considered by Mossong et al. This a a quite reasonable assumption as both Brazil and Portugal have mainly an European culture and most of its population live in cities, as in most Europe. Besides the contact matrix used in the model is obtained properly considering the population by age-group in each country, which results in different contact matrices. The time evolution of the contact structure is approximately taken into account by a constant factor multiplying the contact matrix that considers at the same time the increase or decrease of average contacts and probability of transmission, and fitted from real data, as explained in the text.

COMMENT: "In a study I found that prioritizing adults aged 60+ years remained the best way to reduce mortality and YLL for R0 ≥ 1.3, but prioritizing adults aged 20 to 49 years was superior for R0 ≤ 1.2. Prioritizing adults aged 20 to 49 years minimized infections for all values of R0 investigated. How does this results connect with the results in this manuscript. If differ, why?"

ANSWER: The point here is that if you have to chose only by age-group, then it starting by the group with higher age results in the most significant reduction of hospitalizations and deaths, and this is currently the most frequent strategy adopted by far. What we are drawing some attention on that there are other possibilities that may result in a more significant reduction of cases and deaths by COVID-19, i.e. by first vaccinating the superspreaders group and afterwards by following reverse chronological order of age.

COMMENT: "It would be good if the authors considered two factors more e.g. vaccines with imperfect transmission-blocking effects, incorporation of population seroprevalence and individual serological testing."

ANSWER: This was partially taken into consideration when considering vaccines with an efficiency varying from 0.7 to 0.95. A more detailed study in this direction is underway in out group.

COMMENT: "I found many works in the literature on approximately same topic. In such context, this can be considered a prototype work. But, the model extracted results offered herein this study provoked me to the favor of publishing this manuscript in Plos One. In my opinion, it needs a further review after a major revision where the authors address the above flaws."

ANSWER: We believe that the above mentioned corrections and answers satisfy this referee's comment.
